# CITED1 promotes proliferation of papillary thyroid cancer cells via the regulation of p21 and p27

**DOI:** 10.1186/s13578-018-0256-9

**Published:** 2018-11-06

**Authors:** Hai Li, Hongyu Guan, Yan Guo, Weiwei Liang, Liehua Liu, Xiaoying He, Weijian Ke, Xiaopei Cao, Haipeng Xiao, Yanbing Li

**Affiliations:** Department of Endocrinology and Diabetes Center, The First Affiliated Hospital of Sun Yat-sen University, 58 Zhongshan Road II, Guangzhou, 510080 Guangdong China

**Keywords:** CITED1, Papillary thyroid cancer, Proliferation, p21, p27

## Abstract

**Background:**

It has been reported that CBP/p300-Interacting Transactivator with glutamic acid [E]/aspartic acid [D]-rich C-terminal domain 1 (CITED1) is overexpressed in papillary thyroid cancer (PTC). However, the functional significance and underlying mechanisms of CITED1 in PTC are largely unknown.

**Methods:**

The Cancer Genome Atlas dataset and real-time PCR were used to determine the expression of CITED1 in PTC. The role of CITED1 in PTC cell proliferation was determined conducted using 3-(4,5-dimethylthiazol-2-yl)-2,5-diphenyltetrazolium bromide (MTT), colony formation, 5-ethynyl-2′-deoxyuridine (EdU) incorporation, and flow cytometry assays in vitro, and a subcutaneous xenotransplantation tumor model in nude mice was established to analyze tumor growth in vivo. We studied the potential mechanisms underlying the contribution of CITED1 to PTC proliferation using western blotting and luciferase assays.

**Results:**

We found that CITED1 was highly expressed in PTC. In vitro and in vivo experiments demonstrated that CITED1 was involved in PTC cell proliferation and tumorigenesis. Then, gain- and loss-of-function experiments revealed that CITED1 decreased the expression of p21 and p27, and thereby increased the phosphorylation of pRb as well as E2F1 transcriptional activity.

**Conclusions:**

Our results suggest that CITED1 is overexpressed in PTC and that CITED1 promotes the proliferation of PTC cells via the regulation of p21 and p27, which indicates that CITED1 might be a potential therapeutic target in the treatment of PTC.

**Electronic supplementary material:**

The online version of this article (10.1186/s13578-018-0256-9) contains supplementary material, which is available to authorized users.

## Introduction

The incidence of thyroid cancer, which is the most common endocrine malignancy, has continued to increase worldwide over the past several decades [1, 2]. Thyroid cancer can be classified as follows: follicular epithelial cell-derived papillary thyroid cancer (PTC), follicular thyroid cancer (FTC), and anaplastic thyroid cancer (ATC), and para-follicular C-cell-derived medullary thyroid cancer (MTC). PTC is the most common subtype, and its standard therapies include surgery, radio-iodine treatment, and thyroid-stimulating hormone suppression [3]. Most patients respond well to these treatments, but some cases are progressive with poor clinical outcomes [4]. Molecular genetic studies have shown a series of genetic and epigenetic changes that are involved in thyroid carcinogenesis [5, 6]. Further assessment of the genetic events involved in thyroid cancer initiation and progression will provide new insight into thyroid tumorigenesis and may lead to effective therapeutic strategies.

CREB-binding protein/p300 Interacting Transactivator with glutamic acid [E]/aspartic acid [D]-rich C-terminal domain 1 (CITED1) is located on chromosome Xq13.1 and encodes a 27-kDa nuclear protein belonging to the CITED family of proteins [7]. This protein family co-regulates transcriptional nuclear proteins via their transactivator domains [7–9]. CITED1 was found to be significantly overexpressed in PTC [10–15]. It has been reported that a lower methylation ratio of CpGs in the promoter region of CITED1 is associated with higher expression of CITED1 mRNA, which suggests the involvement of epigenetic regulation in the overexpression of CITED1 in PTC [16]. Study by Schulten et al. implicated that CITED1 might associated with molecular processes of a brain metastasis from a PTC [17]. In addition, CITED1 might be involved in the roles of some miRNAs in PTC [18].

However, the biologic function and the underlying mechanisms of CITED1 in PTC remain largely unknown.

Here, we investigated the role of CITED1 in the progression of PTC and found that CITED1 can promote the proliferation of PTC cells via the regulation of the expression of p21 and p27.

## Materials and methods

### Cell culture

The human thyroid cancer cell lines TPC1 and BCPAP were provided by Prof. Haixia Guan (China Medical University, China). All cell lines were authenticated by short tandem repeat (STR) DNA profiling and were verified to be mycoplasma-free. The cells were cultured in Dulbecco’s modified Eagle’s medium (DMEM) (Gibco, Grand Island, NY) supplemented with 10% fetal bovine serum (FBS; Gibco), 100 U/mL penicillin (Gibco), and 100 mg/mL streptomycin (Gibco) at 37 °C in a 5% CO_2_ humidified incubator.

### Patients and tissue specimens

The cases from which the 12 pairs of fresh PTCs and the adjacent nontumorous thyroid tissues were obtained were diagnosed at the First Affiliated Hospital of Sun Yat-sen University. The fresh tissues were collected and were then frozen and stored in liquid nitrogen until use. The pathology of all specimens was confirmed by two pathologists. All patients provided informed consent, and ethics approval was obtained from the Institutional Research Ethics Committee.

### Vectors and retroviral infection

The CITED1 construct was generated by sub-cloning PCR-amplified full-length human Clorf106 cDNA into pQCXIP (Clontech, Mountain View, CA). The CITED1-shRNAs (TR313896) were purchased from OriGene (Rockville, MD). These plasmids were transfected into PT67 cells (Clontech) using Lipofectamine 3000 (Invitrogen, San Diego, CA). The supernatant was then harvested, passed through a 0.45-μm filter and incubated with the indicated cells along with polybrene (8 μg/mL). Subsequently, stable cell lines were selected with 0.5 μg/mL of puromycin for 2 weeks [19].

### RNA extraction, RT (reverse transcription), and real-time PCR

RNA extraction, RT, and real-time PCR were performed as previously described [20]. The primers selected are as follows: CITED1 forward, 5′-GAATCACTCTCTCCTTCTG-3′ and reverse, 5′-CATCAGCACTTCCTCATC-3′; glyceraldehyde-3-phosphate dehydrogenase (GAPDH) forward, 5′-TTGAGGTCAATGAAGGGGTC-3′ and reverse, 5′-GAAGGTGAAGGTCGGAGTCA-3′.

### Cell viability

Cell viability was determined using a 3-(4,5-dimethyl-thiazol-2-yl)-2,5-diphenyltetrazolium bromide (MTT) colorimetric assay. The cells were seeded at a density of 5 × 10^3^ cells per well in 96-well plates. Then, at the same time on days 1, 2, 3, 4, 5 and 6, the cells were incubated with 20 μL MTT (Sigma-Aldrich, St. Louis, MO) per well for 4 h. The culture medium was removed and 200 μL dimethyl sulfoxide (DMSO) (Amresco, Solon, OH) was added to each well. The plates were then shaken for 30 min, and the optical density (OD) at 490 nm was measured using an ELISA plate reader. Each sample was analyzed three times in triplicate.

### Flow cytometry analysis

The indicated cells were harvested and washed in ice-cold phosphate-buffered saline (PBS), which was followed by fixation in 80% ice-cold ethanol in PBS. After the cells were spun down in a cooled centrifuge and resuspended in cold PBS, RNAase (Sigma-Aldrich) was added at a final concentration of 2 μg/mL; the cells were then incubated at 37 °C for 30 min followed by incubation in 20 μg/mL of propidium iodide (Sigma-Aldrich) for 20 min at room temperature. The analysis was performed using a flow cytometer (Beckman-Coulter, Hialeah, FL).

### EdU incorporation assay

The EdU incorporation assay was performed according to the manufacturer’s protocol (RiboBio, Guangzhou, China) [21]. Briefly, the indicated cells were cultured in triplicate in 24-well plates for 24 h and were then treated with 50 μM of EdU for 2 h at 37 °C. After they were fixed in 4% formaldehyde for 10 min and permeabilized with 0.5% Triton X-100 for 10 min at room temperature, the cells were treated with 1× Apollo reaction cocktail for 30 min. Subsequently, the cell nuclei were stained with Hoechst 33342 and visualized under a fluorescence microscope. Each experiment included three replicates and was performed in triplicate.

### Colony formation assay

For the colony formation assay, cells were plated in 6-well plates at a density of 500 cells per well. The cells were allowed to grow for 10 days at which point they were stained with crystal violet. The plates were imaged, and the numbers of colonies formed by the indicated cells were quantified using the Quantity One software package (Bio-Rad, Hercules, CA). Each experiment was repeated three times.

### Western blotting

Western blotting was performed according to a standard method as previously described [20]. The antibodies used for immunoblotting were as follows: anti-CITED1, anti-cyclin B1, anti-cyclin D3, anti-cyclin A2, anti-cyclin D1, anti-cyclin E1, anti-CDK4, anti-CDK6 (Abcam, Cambridge, MA), anti-cyclin D2, anti-CDK2 (BD Pharmingen, San Diego, CA), anti-cyclin E2, anti-p21Cip1, anti-p27Kip1 (Cell Signaling Technology, Beverly, MA), and anti-α-tubulin (Sigma-Aldrich, St. Louis, Missouri). The bands were quantified using Quantity One software (Bio-Rad, Hercules, CA).

### In vivo experiments

Five female BALB/c mice (4 weeks of age) were used to assess the effect of CITED1 on tumor growth in vivo. Briefly, 1 × 10^7^ of the indicated cells were injected subcutaneously into the dorsal flank of each mouse. Tumor size was measured every 5 days, and the tumor volume was estimated. Thirty days after the injection, the mice were euthanized, and the tumors were removed and weighed. All experiments that involved the use of animals were conducted in accordance with the recommendations in the Guide for the Care and Use of Laboratory Animals of the National Institutes of Health. The protocol was approved by the Institutional Animal Care and Use Committee of Sun Yat-sen University.

### Reporter assay

Dual-luciferase reporter assays (Promega, Madison, WI) were performed according to the manufacturer’s instructions as previously described [15]. Briefly, the indicated cells were seeded in triplicate in 24-well plates and were allowed to settle for 24 h. The cotransfection of pE2F-TA-Luc plasmid (Clontech, San Francisco, CA) and 1 ng pRL-TK Renilla was performed using Lipofectamine 2000 Reagent (Life Technologies, Gaithersburg, MD) according to the manufacturer’s protocol. Thirty-six hours after transfection, the cells were harvested and lysed, and the luciferase activity was assessed. The firefly luciferase activity was normalized to that of Renilla luciferase. Three independent experiments were performed.

### Statistical analysis

All data are presented as the means ± standard deviations (SDs) of at least three independent experiments. Statistical analysis was performed using SPSS17.0 software (SPSS Inc., Chicago, IL), and the Student t-test was used to compare the differences between two groups. P < 0.05 was considered significant.

## Results

### CITED1 is upregulated in human PTC

**Fig. 1:**
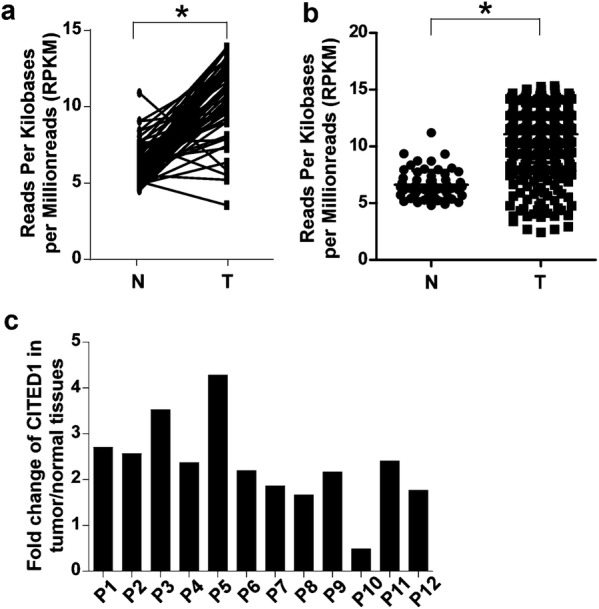
CITED1 is upregulated in human PTC. **a** The expression of CITED1 in 59 pairs of primary PTC (T) versus corresponding non-cancerous thyroid tissues (N) using RNAseq data sets deposited in the TCGA. **b** CITED1 expression in 496 PTC specimens and 59 non-cancerous thyroid specimens in the TCGA dataset. **c** Expression of CITED1 in 12 paired tumors and adjacent nontumorous thyroid tissues assessed by qRT-PCR. *P < 0.05

Initially, we analyzed the expression of CITED1 in 59 pairs of thyroid tumor specimens and corresponding adjacent noncancerous thyroid tissues using the thyroid cancer RNAseq data deposited in the TCGA. As shown in Fig. 1a, the expression level of CITED1 was significantly elevated in most PTC tissues compared with their paired adjacent noncancerous thyroid tissues. Moreover, CITED1 was significantly upregulated in PTC tissues (n = 496) compared with non-cancerous thyroid tissues (n = 59) in the TCGA cohort (Fig. 1b). Next, we confirmed the expression of CITED1 in the 12 pairs of PTC and adjacent noncancerous thyroid tissues using quantitative RT-PCR. As shown in Fig. 1c, CITED1 expression was elevated in tumor tissues compared with corresponding noncancerous tissues.

### Silencing of CITED1 inhibits cell proliferation

**Fig. 2:**
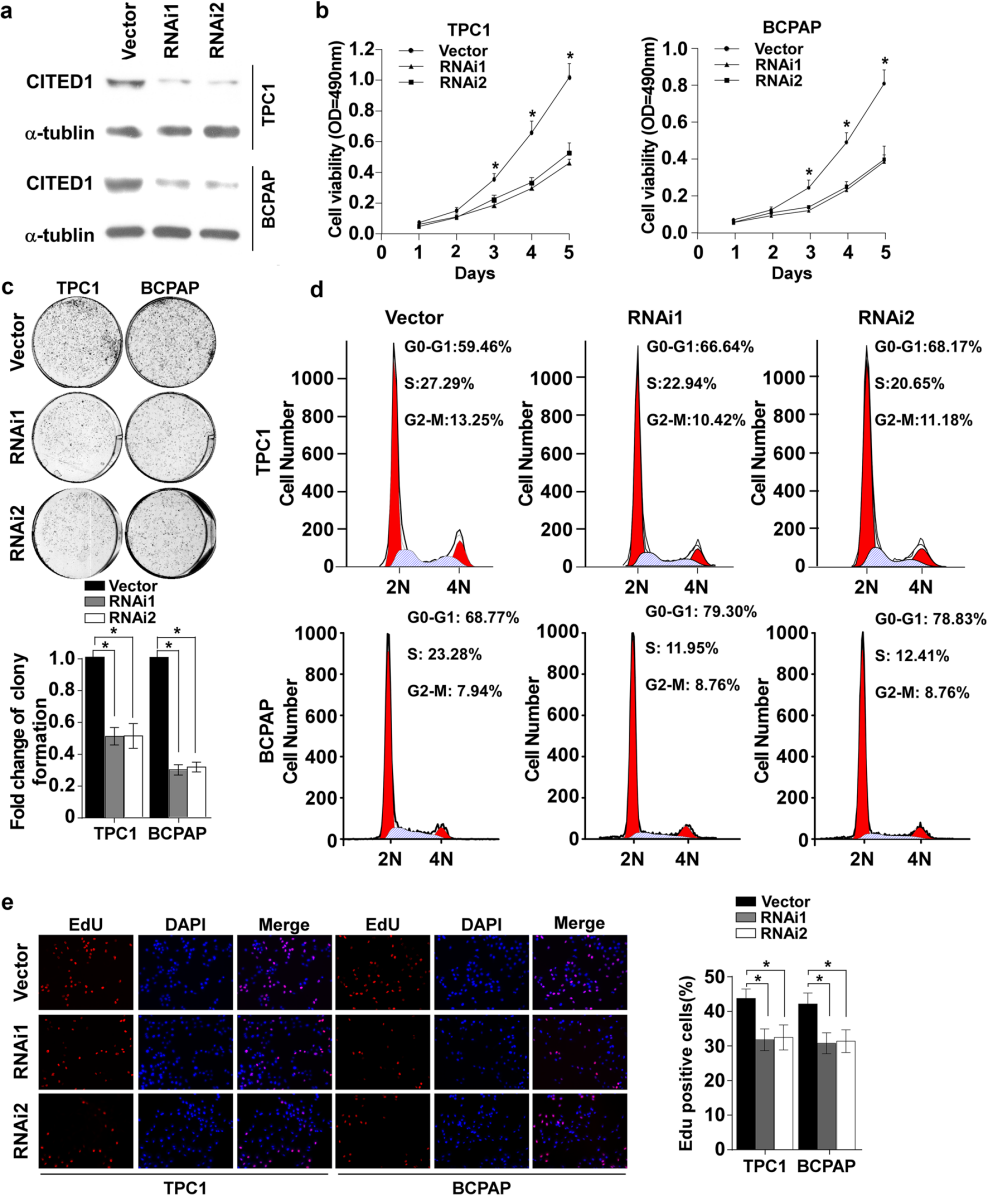
Silencing of CITED1 inhibits cell proliferation. **a** Protein expression of CITED1 in the indicated cells was analyzed by western blotting analysis. α-Tubulin was used as the loading control. **b** An MTT assay was performed to investigate the effect of CITED1 on the viability of the indicated PTC cells at the indicated time points. **c** Representative micrographs and relative quantification of colony formation assays of the indicated cells. **d** Flow-cytometric determination of the proportion of the studied cells in distinct cell-cycle phases. Knockdown of CITED1 increased the proportion of cells in G1 phase. **e** Representative images and relative quantification of EdU incorporation assays. For **c** and **e**, the data are reported as the mean ± SD of three independent experiments. *P < 0.05

To investigate the functional role of CITED1 in PTC progression, TPC1 and BCPAP cells in which CITED1 was stably knocked down were established (Fig. 2a and Additional file 1: Fig. S1a). Next, we evaluated cell viability by MTT assay. As shown in Fig. 2b, the silencing of CITED1 significantly decreased the rate of cell growth compared with control cells. Furthermore, the colony formation ability of the cells after depletion of CITED1 was significantly inhibited compared with that of control cells (Fig. 2c). To investigate the effect of CITED1 on cell cycle progression in PTC cells, fluorescence-activated cell sorting analyses were performed. As shown in Fig. 2d, depletion of CITED1 increased the proportion of cells in G1 phase. Moreover, an EdU incorporation assay was performed, and the results showed that the proportion of EdU-positive cells was significantly decreased in CITED1-silenced cells (Fig. 2e). Taken together, these data demonstrated that the knockdown of CITED1 inhibited PTC cell proliferation.

### Ectopic overexpression of CITED1 promotes proliferation of PTC

**Fig. 3:**
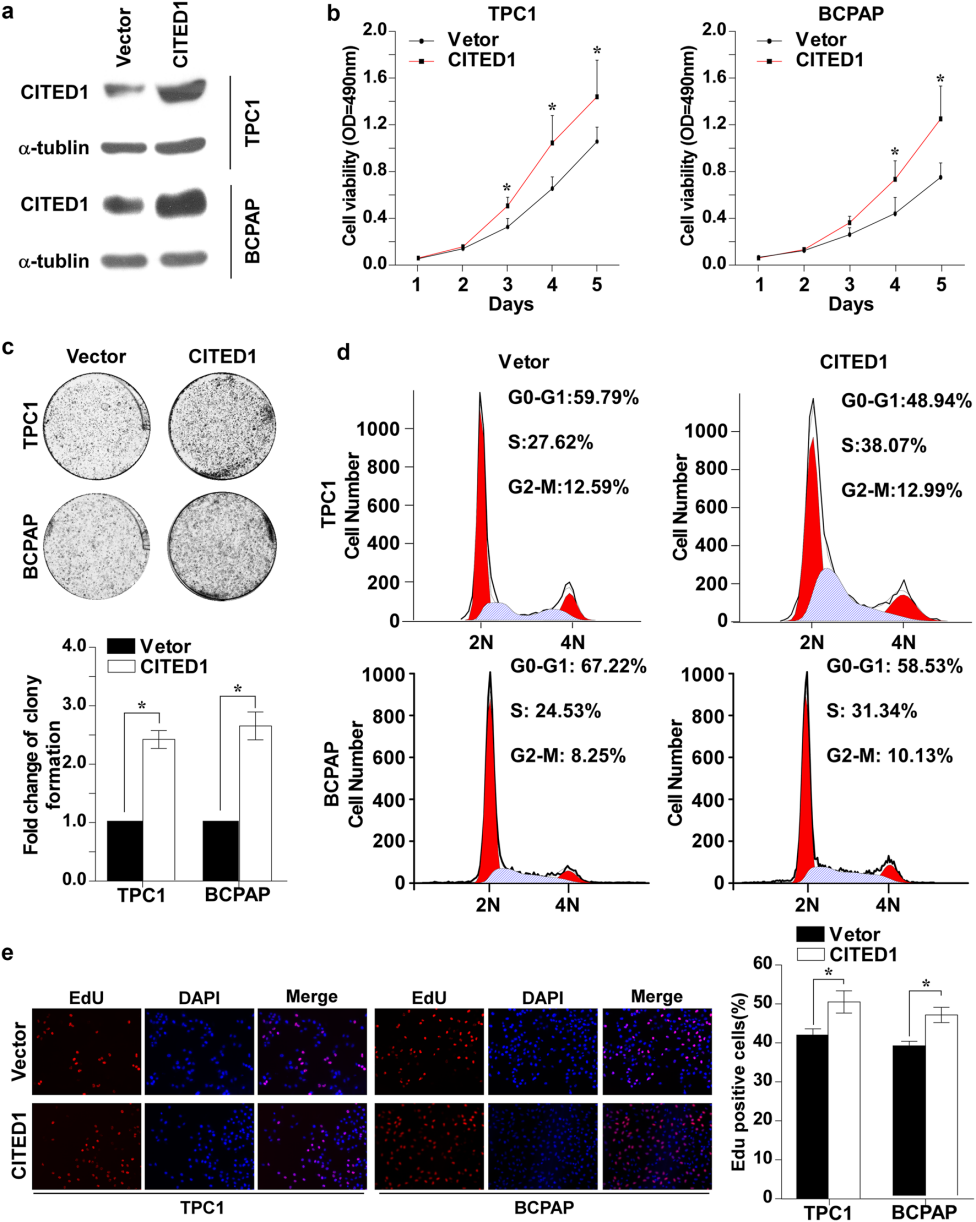
CITED1 promotes proliferation of PTC cell lines. **a** Overexpression of CITED1 was confirmed in the studied cells (TPC-1 and BCPAP) by western blotting analysis. α-tubulin was used as the loading control. **b** MTT assays were conducted to examine the viability of the indicated cell lines. **c** Representative images (upper panel) and relative quantification (lower panel) of the colony formation assay results. **d** Proportions of cells in distinct cell-cycle phases in the indicated cell lines were determined by flow cytometry. Overexpression of CITED1 decreased the proprotion of cells in G1 phase. **e** Representative micrographs (left panel) and quantification (right panel) of the EdU incorporation assay results. For **b**, **c**, and **e**, the results derived from three independent experiments and are expressed as the mean ± SD. *P < 0.05

To further confirm the role of CITED1 in PTC cell proliferation. TPC1 and BCPAP cells in which CITED1 was overexpressed were established (Fig. 3a and Additional file 1: Fig. S1b). As shown in Fig. 3b, the overexpression of CITED1 significantly increased cell viability compared with the vector-control. Furthermore, the colony formation ability was significantly promoted by CITED1 overexpression (Fig. 3c). As shown in Fig. 3d, flow cytometry analysis revealed that ectopic overexpression of CITED1 increased the proportion of cells in S phase. Moreover, the EdU incorporation assay showed that the proportion of EdU-positive cells was significantly increased in cells with forced expression of CITED1 (Fig. 3e). Taken together, these data demonstrated that ectopic overexpression of CITED1 promoted PTC cell proliferation.

### Downregulation of CITED1 suppresses tumor growth in vivo

**Fig. 4:**
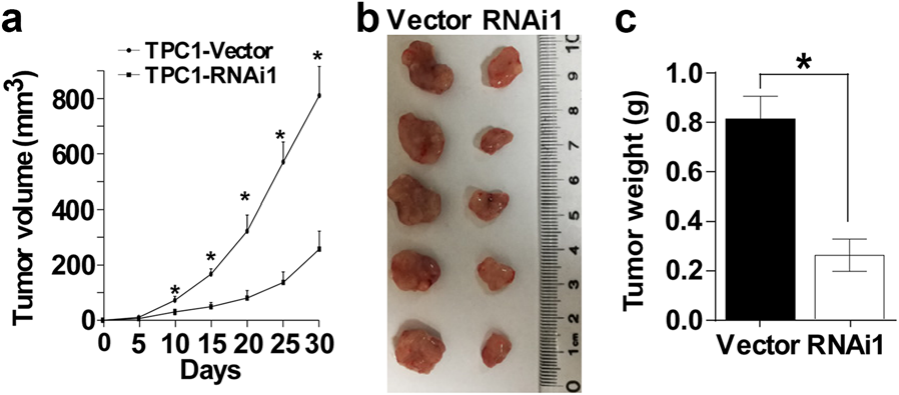
Downregulation of CITED1 suppresses tumor growth in vivo. **a** Quantitative analysis of tumor volumes. **b** Image of subcutaneous tumors isolated from nude mice. **c** Quantitative analysis of tumor weights. The indicated tumor volumes and weights represent the mean ± SD of five animals in each group. *P < 0.05

To investigate the biological effect of CITED1 silencing on tumor growth in vivo, we proceeded with the establishment of a subcutaneous xenograft tumor model in nude mice. The growth curve revealed a dramatic decrease in tumor growth in the group in which CITED1 was knocked down (Fig. 4a). Moreover, the tumor size (Fig. 4b) and weight (Fig. 4c) of the group in which CITED1 was silenced were lower than the tumor size and weight of the control group. These data further confirmed the role of CITED1 in PTC cell proliferation.

### Silencing of CITED1 elevates the expression of the CKIs p21 and p27

**Fig. 5:**
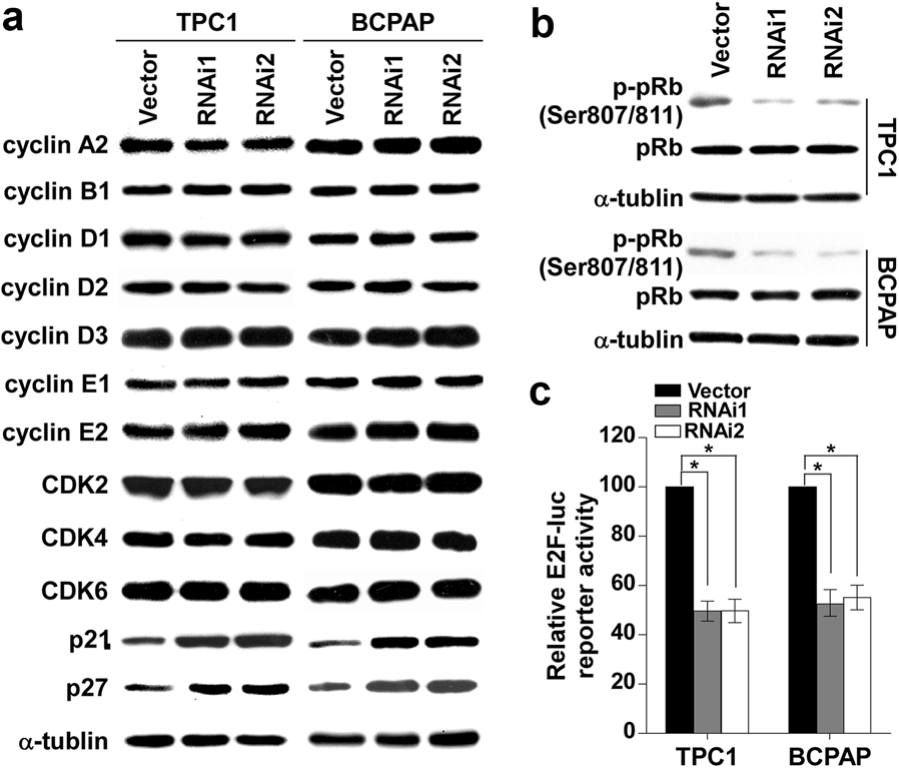
Knockdown of CITED1 upregulates the expression of the CKIs p21 and p27. **a** Western blotting analysis was performed to detect the expression levels of the cell cycle regulators cyclin A2, cyclin B1, cyclin D1, cyclin D2, cyclin D3, cyclin E1, cyclin E2, CDK2, CDK4, CDK6, p21^Cip1^, and p27^Kip1^ in the indicated cells. α-tubulin was used as the loading control. **b** Silencing of CITED1 in the studied cells significantly inhibited the phosphorylation of pRb at the Ser608 residue. α-tubulin served as the sample loading control. **c** Silencing of CITED1 attenuated E2F transcriptional activity according to the E2F-luc reporter assay. The results are derived from three independent experiments and are expressed as the mean ± SD. *P < 0.05

To delineate the mechanisms underlying the effect of CITED1 on PTC cell proliferation, western blot analysis was performed to assess the protein expression level of cell cycle regulators. As shown in Fig. 5a and Additional file 1: Fig. S1c, the knockdown of CITED1 increased the expression levels of p21 and p27 proteins in TPC1 and BCPAP cells, but no changes in the protein levels of cyclin A2, cyclin B1, cyclin D1, cyclin D2, cyclin D3, cyclin E1, cyclin E2, CDK2, CDK4, or CDK6 were observed. We further examined the effects of CITED1 silencing on pRb phosphorylation and E2F transcriptional activity. As shown in Fig. 5b, c, and Additional file 1: Fig. S1d, depletion of CITED1 decreased the phosphorylation of pRb and E2F transcriptional activity. Taken together, these results indicated that CITED1 suppressed proliferation through the upregulation of p21 and p27.

### Overexpression of CITED1 decreases the expression of CKI p21 and p27

**Fig. 6:**
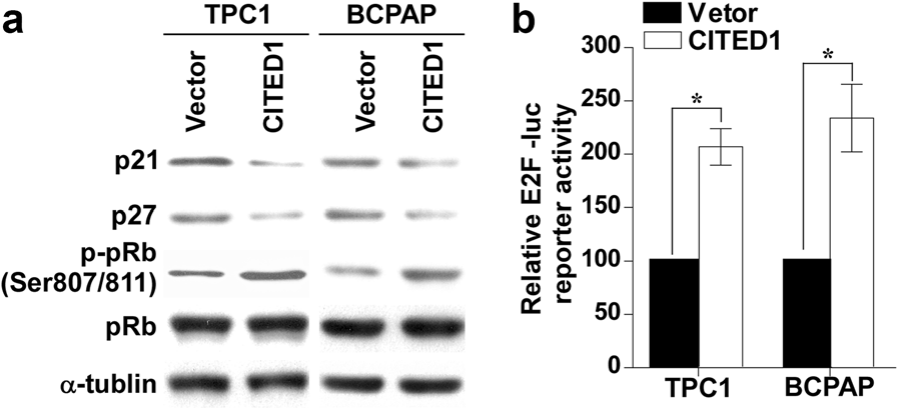
CITED1 overexpression inhibits the levels of p21 and p27. **a** Western blotting analysis was performed to detect the expression levels of p21 and p27, as well as the phosphorylation of pRb. α-tubulin was used as the loading control. **b** Transcriptional activity of E2F was analyzed by luciferase reporter assays. pRL-TK was used to normalize the luciferase activity. For **b**, data from three independent experiments are shown as the mean ± SD. *P < 0.05

As shown above, the knockdown of CITED1 increased the expression levels of p21 and p27 proteins, decreased the phosphorylation of pRb and inhibited E2F transcriptional activity in TPC1 and BCPAP cells. We further examined the effects of CITED1 overexpression on PTC cells. As shown in Fig. 6a, b and Additional file 1: Fig. S1e, f, ectopic overexpression of CITED1 decreased the expression of p21 and p27 proteins, but increased the phosphorylation of pRb and E2F transcriptional activity. Taken together, these results further suggested that p21 and p27 mediated the effect of CITED1 on PTC cell proliferation.

## Discussion

CITED1 is the central member of the CITED family of transcriptional co-regulators and was originally cloned from a differential display screen between pigmented mouse B16 melanoma cells and their dedifferentiated weakly pigmented derivative, B16F10 cells [7]. Studies have demonstrated that CITED1 is a non-DNA binding nuclear transcriptional co-regulator capable of influencing transcription downstream of the TGFβ, ERα, and Wnt/β-Catenin genes. These effects are dependent on the conserved CITED family CR2 domain, which stimulates CITED1-CBP/P300 binding. Moreover, CITED1 is believed to act by stabilizing the CBP/P300-ERα interaction. In some cases, this protein represses the transcription of β-Catenin by competing with CBP/P300 transcriptional co-activators [9, 22–24].

Notably, accumulating evidence has shown that CITED1 plays critical roles in cancer pathogenesis [25–30]. Several studies have demonstrated that CITED1 is significantly overexpressed in PTC tissues [10–15]. The results of the current study also showed that CITED1 was overexpressed in clinical PTC specimens, which is consistent with previous studies, and indicated that CITED1 may play an important role in the development and progression of PTC. Indeed, our in vitro and in vivo experiments strongly demonstrated the pro-proliferative role of CITED1 in PTC cells. These results further demonstrated the oncogenic role of CITED1.

In the additional studies that aimed to delineate the underlying mechanisms of CITED1 in PTC cells, we found that two important cell cycle-related genes, p21 and p27, were regulated by CITED1. p21 (also known as *p21*^*Waf1/Cip1*^, encoded by CDKN1A) is a potent cyclin-dependent kinase inhibitor that interacts with and inhibits the activity of cyclin-CDK2, -CDK1, and -CDK4/6 complexes. p21 also functions as a regulator of cell cycle progression during G1 and S phases. It has been reported that p21 is the target of the tumor suppressor protein p53 and its isoform [31, 32], and thus functions as a tumor suppressor in a variety of cancer types [33]. p27 (also known as KIP1, encoded by CDKN1B) is an atypical tumor suppressor that regulates G0 to S phase transition by binding to and regulating the activity of CDK1 and CDK2. In G0 and early G1, p27 translation and protein stability are maximal, and it binds and inhibits the cyclin E-CDK2 complex [34, 35]. Our data demonstrated that p21 and p27 expression levels were increased and that these proteins were involved in the pro-proliferative effect of CITED1 in PTC cells. These data have provided new insights into the role of CITED1 in the development and progression of malignancies.

In conclusion, these data suggest that CITED1 is overexpressed in PTC tissues. Knockdown of CITED1 inhibits the proliferation of PTC cells, while overexpression of CITED1 promotes the proliferation of PTC cells, and these effects may via regulating p21 and p27. These findings may provide new insights into potential targeted therapies in the treatment of PTC.

## Additional file

**Additional file 1.** **a**–**f** Quantification of indicated band densities using Quantity One software (Bio-Rad, Hercules, CA).

## Abbreviations

ATC: anaplastic thyroid cancer
CITED1: CBP/p300-InteractingTransactivator with glutamic acid [E]/aspartic acid [D]-rich C-terminal domain 1
DMEM: Dulbecco’s Modified Eagle’s Medium
FBS: fetal bovine serum
FTC: follicular thyroid cancer
GAPDH: glyceraldehyde-3-phosphate dehydrogenase
MTC: medullary thyroid cancer
PTC: papillary thyroid cancer
RT-PCR: reverse transcription-polymerase chain reaction
TCGA: The Cancer Genome Atlas
WB: western blotting
