# Supplementary figures and images for: CITED1 promotes proliferation of papillary thyroid cancer cells via the regulation of p21 and p27
Source: Cell Biosci. 2018 Nov 6;8:57. doi: 10.1186/s13578-018-0256-9 (PMC6219258; doi:10.1186/s13578-018-0256-9)

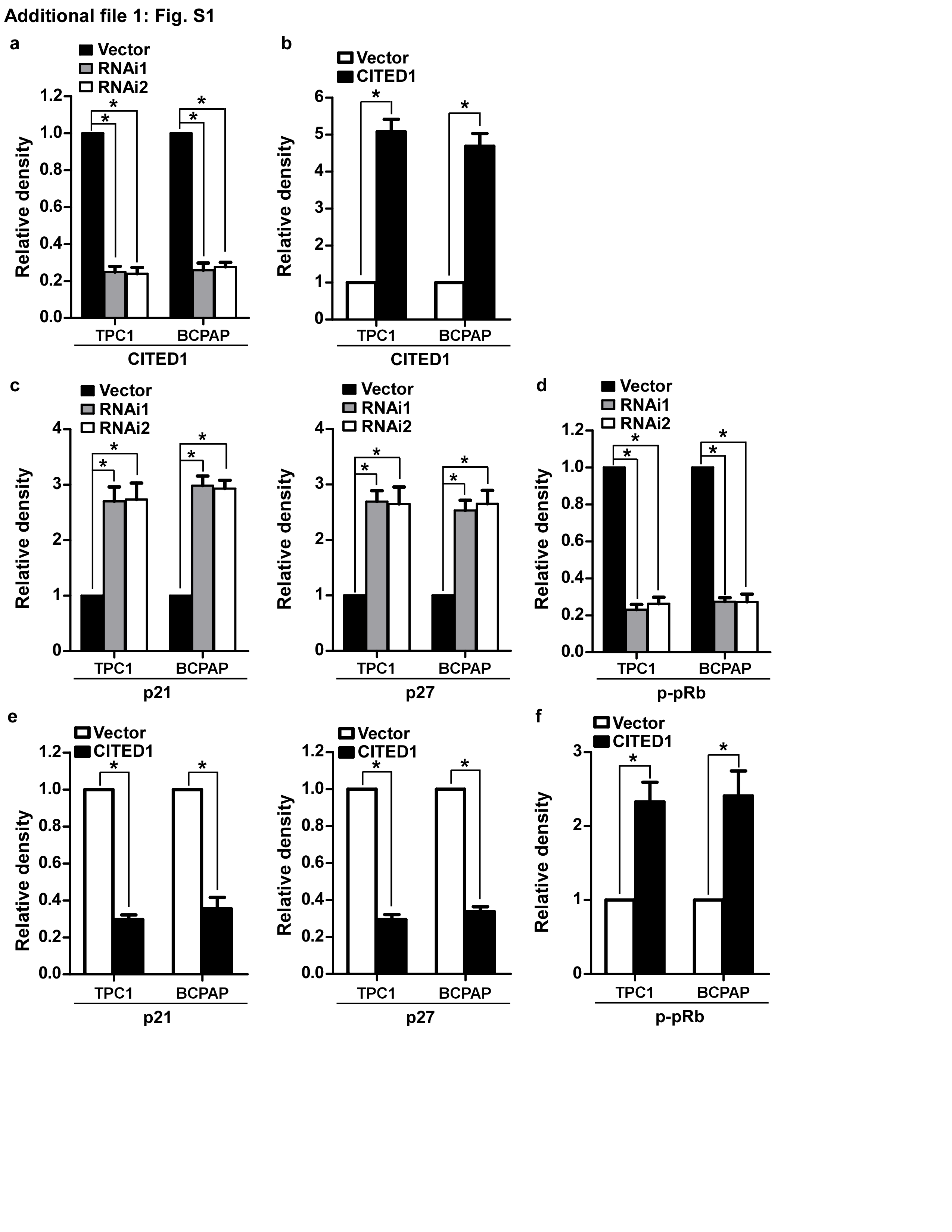

Supplement: Supplementary file 1 — Additional file 1. a–f Quantification of indicated band densities using Quantity One software (Bio-Rad, Hercules, CA). [file 13578_2018_256_MOESM1_ESM.tif]
